# Draft Genome Sequences of *Kosmotoga* sp. Strain DU53 and *Kosmotoga arenicorallina* S304

**DOI:** 10.1128/genomeA.00570-16

**Published:** 2016-06-16

**Authors:** Stephen M. J. Pollo, Rhianna Charchuk, Camilla L. Nesbø

**Affiliations:** a Department of Biological Sciences, University of Alberta, Edmonton, Alberta, Canada; b Centre for Ecological and Evolutionary Synthesis, Department of Biology, University of Oslo, Oslo, Norway

## Abstract

Here, we announce the draft genome sequences of two thermophilic *Thermotogae* bacteria: *Kosmotoga* sp. strain DU53, isolated from a continental oil reservoir, and *Kosmotoga arenicorallina*, isolated from hydrothermal sediments. The sequences will provide further insight into evolution of the *Kosmotogales*.

## GENOME ANNOUNCEMENT

Members of the genus *Kosmotoga* are anaerobic thermophilic bacteria isolated from oil reservoirs and hydrothermal environments (1, 2). The type species, *Kosmotoga olearia*, has an extraordinary wide growth temperature range of 20 to 79°C, and its genome was previously sequenced (3). The closest relative of *Kosmotoga* is the only mesophilic *Thermotogae* lineage, *Mesotoga* (4). This, together with its wide temperature growth range, makes these bacteria well suited for studying thermal adaptations (5).

Here, we present draft genome sequences of *Kosmotoga arenicorallina* S304 and *Kosmotoga* sp. strain DU53. *K. arenicorallina* S304 was isolated from hydrothermal sediments with a temperature of ~40°C (2) and purchased from DSMZ (https://www.dsmz.de/). *Kosmotoga* sp. DU53 was isolated from free-water-knockout (FWKO) water collected from oil field D (*in situ* temperature, ~50°C) in Alberta, Canada (6, 7) (available upon request from C.L.N.). Briefly, bottles containing 50 ml of *Kosmotoga olearia* medium (1) were inoculated with 2 ml of FWKO water that had been stored anoxically at room temperature (RT) for 4 years, incubated at 55°C for 5 days, and then stored for 5 weeks at RT. Dilution series and bottle plates were made as described by Dipippo et al. (1) and incubated at 55°C for 3 weeks. One white round colony confirmed to be a *Kosmotoga* bacterium by 16S rRNA PCR was selected for genome sequencing.

DNA was extracted from 50-ml cultures of *K. arenicorallina* S304 and *Kosmotoga* sp. DU53, according to the protocol described by Charbonnier and Forterre (8). The purity and quantity of the DNA were measured using NanoDrop and Qubit instruments (Thermo Fisher Scientific). *Kosmotoga* sp. DU53 DNA was sheared using the Ion Shear Plus kit, and a library was constructed using the Ion Plus fragment library kit and sequenced on an Ion Torrent PGM (all from Life Technologies) using a 316 D Chip and 500 flows. The *K. arenicorallina* S304 library was constructed using the Nextera XT kit and sequenced as one of 10 pooled barcoded libraries on a MiSeq (all from Illumina) using 500 cycles generating 2 × 250-bp paired-end reads.

The genome of *K. arenicorallina* S304 was assembled *de novo* by CLC Genomics Workbench 7.0.4, using trimming settings, automatic word size, a bubble size corresponding to the average length of the input reads, a minimum contig length of 1,000 bp, and reads mapped back to the contigs. Four contigs containing parts of two nonidentical 16S genes were located and manually resolved using its published 16S gene sequences (accession numbers AB530678 and AB530679). This resulted in 40 contigs totaling 2,113,627 bp, with an *N*_50_ of 109,886 bp, a longest contig size of 350,318 bp, and a G+C content of 41.0%.

*De novo* assembly of the *Kosmotoga* sp. DU53 genome was done using MIRA 3, with Ion Torrent settings (9) (http://sourceforge.net/projects/mira-assembler/), resulting in 97 contigs totaling 2,375,260 bp, with an *N*_50_ of 66,806 bp, a longest contig size of 221,738 bp, and a G+C content of 41.4%.

Both draft genomes were annotated in the NCBI Prokaryotic Genome Automatic Annotation Pipeline (PGAAP [10]), which identified 2,038 genes and 1,980 coding sequences (CDSs) for *K. arenicorallina* S304 and 2,504 genes and 2,430 CDSs for *Kosmotoga* sp. DU53.

### Nucleotide sequence accession numbers.

Both whole-genome shotgun projects have been deposited at DDBJ/EMBL/GenBank under the accession numbers JFHK00000000 and JGCK00000000 for *Kosmotoga arenicorallina* S304 and *Kosmotoga* sp. DU53, respectively. The versions described in this paper are the first versions, JFHK01000000 and JGCK01000000.
